# Screen Printable Sol-Gel Materials for High-Throughput Borosilicate Glass Film Production

**DOI:** 10.3390/molecules27175408

**Published:** 2022-08-24

**Authors:** Jonas D. Huyeng, Raphael Efinger, David Bruge, Oliver Doll, Roman J. Keding, Florian Clement

**Affiliations:** 1 Fraunhofer Institute for Solar Energy Systems ISE, Heidenhofstr. 2, 79110 Freiburg, Germany; 2 Merck KGaA, Frankfurter Str. 250, 64293 Darmstadt, Germany

**Keywords:** sol-gel, borosilicate, screen printing, solar cells, rheology, doping

## Abstract

The use of sol-gel materials can simplify the industrial fabrication of high-efficiency silicon solar cells if a suitable deposition method is established. In this work, we investigate the possibilities to adapt a borosilicate glass sol-gel to provide a stable screen printing process. This material has previously been used as a boron dopant source for silicon solar cells. We now use an adjusted synthesis process, with an increased gelling time and different additives. This changes the rheological properties (i.e., the elastic and viscous moduli *G′* and *G″*) in a way that avoids the dripping of paste through the screen and that stabilizes the material transfer in subsequent printing steps. Using this synthesis process, we were able to show a printing process with long-term stability of more than 500 prints. When comparing the adjusted to the initial paste, we show that, after thermal treatment, the obtained thin films are very similar in terms of their constitution, with a refractive index between *n* = 1.47 (initial) and *n* = 1.55 (adjusted). We also show that they provide the same amount of doping under the tested conditions (950 °C, 30 min), resulting in sheet resistances of *R*_□_ = (42.5 ± 2.6) Ω/□ (initial) and *R*_□_ = (46.4 ± 3.6) Ω/□ (adjusted).

## 1. Introduction

Sol-gel materials are known to create high-quality films of different constitution upon using the right synthesis and processing parameters. The liquid state of the sol-gel material makes it very flexible in terms of applications and it is also attractive for additive printing. In photovoltaic (PV) research, sol-gel materials have been suggested for use in anti-reflective coatings [1], dielectric passivation layers [2] or, recently, as dopant sources [3]. In most cases, the application technology of choice is spin-on deposition. While it is a flexible choice for all kinds of different sol-gel materials, it has a clear scaling issue in the light of PV mass manufacturing based on silicon substrates. Here, the processing costs need to be minimized in order to reduce the costs per device. In the past decades, this has been realized by the optimization of throughput and by the use of reliable, but inexpensive, tools such as screen printing. Another factor, which affects the costs of generated electricity from solar cells, is the photo conversion efficiency [4]. The solar cell structure, which allows for the highest efficiency for a single-junction silicon solar cell, is the so-called “interdigitated back-contact” (IBC) structure [3,5,6]. Here, all contacts are placed on the rear side, thus eliminating optical reflections on the front side. However, to realize the interdigitated patterns, several structuring steps have to be implemented during the manufacturing process.

### 1.1. Screen Printing of Functional Layers

Flatbed screen printing was suggested for silicon solar cell fabrication as early as 1975 [7]. To fabricate the electrodes, metal pastes (e.g., Ag, Al) can be applied in the desired shape on the front and on the rear side of the cell. Besides the metal component, such pastes typically consist of solvents and binders to adjust the rheology. Additionally, glass frit components can be added to induce a local etching of dielectric layers for contact formation with the underlying silicon [8]. The industrial metallization step is almost exclusively done via screen printing today [9].

Naturally, screen printing can also produce other structured functional layers. To design a suitable paste, the process dynamics and the necessary rheological requirements have to be considered. The screens used for printing consist of a fine mesh of wires and a structured emulsion film to realize the desired pattern. During the printing process, the paste is first pushed across the screen by the flood bar which covers the whole cell area. Typical pastes are shear thinning and therefore easily flow into the openings of the emulsion film. Afterwards, the squeegee pushes onto the screen with a defined pressure that leads to a deformation of the screen. The paste in the emulsion openings touches the substrate surface and sticks to it. When the squeegee moves forward, the screen snaps back to its original position and the paste is ejected from the screen. The rheology of the paste at rest (i.e., without external stress) determines how far the printed structures flow on the surface.

To develop a suitable paste for screen printing, it is therefore important to have a rather high-viscosity liquid that does not drip through the mesh, that is ideally shear thinning to improve the transfer of paste to the substrate, and that shows a limited flow on the substrate surface after printing.

### 1.2. Liquid Dopant Sources for Silicon Solar Cells

To utilize photoconversion in silicon, a *p*-*n* junction of oppositely doped semiconductor regions can be used [10,11]. This is mostly realized by impurity diffusion of a suitable dopant species, e.g., B or Ga for *p*-type doping and P or As for *n*-type doping. The impurities are provided in a reservoir of high concentration at the wafer surface and diffuse into the Si bulk in a diffusion furnace at elevated temperatures [12,13].

The dopant reservoir can be grown in situ on the silicon surface as a doped silicon oxide using, for example, BBr_3_ or POCl_3_, resulting in a borosilicate glass (BSG) or a phosphosilicate glass (PSG). Alternatively, a dopant reservoir can be pre-deposited using, for example, plasma-enhanced chemical vapor deposition (PECVD) with SiH_4_, N_2_O, and B_2_H_6_ for BSG [14,15]. Another option is the pre-deposition and drying of a liquid dopant source [16,17,18,19,20,21]. For all dopant sources, it is essential to have a high purity of all precursors. Contaminants, such as metallic impurities, can drastically reduce the lifetime of excited charge carriers inside the silicon by defect recombination, limiting the solar cell performance.

Therefore, the sol-gel synthesis of liquid dopant sources is a promising approach to create a high-purity glass precursor whose rheology can be adjusted by the (poly)condensation step and by using non-contaminating additives.

### 1.3. Scope of this Work

In previous work, we have shown that, in combination with screen printing, a sol-gel-derived BSG can be used as a cost-effective dopant source for IBC solar cells [3]. This process can be used to avoid costlier deposition (e.g., PECVD). Due to the use of screen printing, the layer can also be directly structured and does not require additional masking and etching. However, for mass manufacturing it needs to be ensured that the BSG layer deposition is reliable and stable. In this work, we show how an adopted paste synthesis can help to improve reliability while maintaining the functional properties of the derived layers.

Two recipes for synthesis and post-synthesis adoption have been developed. The first recipe makes only use of the polycondensation step to increase the viscosity of the sol-gel component. Afterwards, the initial solvents are replaced by solvents with high boiling points to increase the material stability during printing in a room temperature ambient air atmosphere. The second recipe features a prolonged synthesis, another solvent system, and termination of the gelling by including sequestrants. These modifications are used to improve the printing performance of the material. Further details are listed in Section 2. We refer to these materials as “BSG-pastes”.

First, the rheology of the two BSG-pastes was investigated in terms of suitability for screen printing in mass production. Afterwards, the pastes were thermally dried and their thin-film properties were analyzed. The achievable dopant levels on silicon wafers were tested using common tube-furnace diffusion and electrical characterization.

## 2. Materials and Methods

### 2.1. Synthesis of Sol-Gel Derived Screen Printing Pastes

The sol-gel synthesis was performed using ultra-high purity precursors (Sigma-Aldrich Chemie GmbH, Taufkirchen, Germany; Merck KGaA, Darmstadt, Germany; Alfa Aesar, Lancashire, United Kingdom). Different alkylalkoxysilanes were tested and mixed with di-boroxane to achieve stable materials that could be dried after screen printing without cracking. After an initial boiling for several hours, solvents with low boiling points are removed and replaced by solvents with high boiling points to improve the stability of the printing process at room temperature. For the advanced BSG-paste, a sequestrant is added to reduce the polycondensation of the sol-gel material and to improve the shelf-life of the material. Afterwards, wax is mixed into the advanced sol-gel material and dispersed for several hours to form a uniform paste. The details of the synthesis procedure were developed and patented by Merck KGaA (Darmstadt, Germany).

### 2.2. Rheological Characterization and Printing Tests

The rheology of the pastes was analyzed using an RS1 rheometer from Thermo-Haake (Waltham, MA, USA) with a cone-plate geometry. Printing was performed using an inline screen printer from ASYS. Screens with a wire diameter of 18 µm and a mesh count of 400 were used. The paste was printed using an RKS carbon squeegee, with a printing pressure of 60 N, and a snap-off distance of 2.0 mm. The printing speed was varied to achieve different paste transfers. This was manually measured using a scale by regularly measuring the wet deposition after several prints. For long-term printing tests, additional paste was added at around every 100th print to replenish the paste reservoir.

### 2.3. Spectroscopic Thin-Film Analysis

For spectroscopic analysis, the pastes were printed onto polished silicon float-zone wafers and dried on a hotplate at different temperatures for 4 min. Spectral ellipsometry was done using an M-200F from J.A. Woollam Co. (Lincoln, Nebraska) at three angles (60°, 70°, 75°). To obtain the refractive index, the data was fit using the “CompleteEASE” software from Woollam. For the BSG layer, a Cauchy model was used, neglecting fitting parameter “*C*”, as suggested in the literature. Fourier-transform infrared (FTIR) spectroscopy was done using a Bruker (Rosenheim, Germany) Vertex80v spectrometer in transmission mode. The reference signal of an unprinted silicon piece was subtracted, and the transmission spectrum was then converted to yield the wavelength-dependent absorbance.

### 2.4. Evaluation of Dopant Properties

To analyze the doping properties, the paste was printed on silicon Czochralski wafers and dried on a hotplate at 450 °C for 4 min. For inductively coupled plasma optical emission spectroscopy (ICP-OES), smaller samples were cut from the wafers and the thin film was dissolved in hydrofluoric acid and diluted in de-ionized water afterwards. The samples used for dopant profiles were loaded into a tube furnace from centrotherm (Blaubeuren, Germany). The wafers were first exposed to a temperature of 800 °C for 15 min, and then they were exposed to a temperature of 950 °C for 30 min. During this time, the furnace was flooded with a mixture of nitrogen and oxygen gas. The oxygen gas flow was nominally set to about 20% of the gas flow of nitrogen. Afterwards the wafers were etched in hydrofluoric acid (concentration of 20%) to remove the thin films and any oxides grown. Electrochemical capacitance-voltage (ECV) measurements were recorded on a CVP21 tool by WEP (Furtwangen, Germany) and converted into a dopant profile using the software provided by the company. Before the ECV measurements, the sheet resistance was checked in a four-point probe measurement using a TLMscan tool from PVtools (Waldburg, Germany).

## 3. Results

### 3.1. Rheology and Suitability for Screen Printing

**Figure 1:**
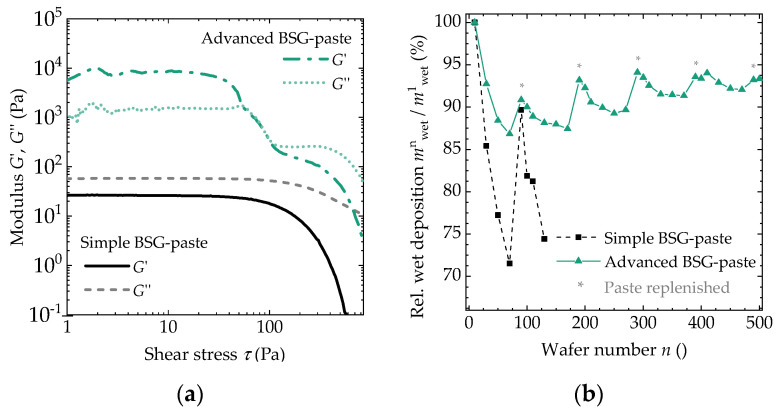
Rheological analysis and printing performance of different pastes. (**a**) Amplitude sweep of shear stress *τ*, showing the change of elastic and viscous moduli *G′* and *G″*, respectively; (**b**) printing test of different pastes. The relative amount of paste transferred *m*_wet_ is measured in subsequent prints. Additional paste is added after 100 prints to maintain a sufficient paste reservoir (marked by a star “*”).

An amplitude sweep of the shear stress τ reveals the evolution of the elastic and viscous moduli *G′* and *G″*, respectively [22]. For the simple BSG-paste, *G″* exceeds *G′* under all conditions (Figure 1a). For higher values of *τ*, both moduli decrease, but *G″* is still higher than *G′*. When measuring the BSG-paste transfer in screen printing (wet deposition m_wet_), we see that the transferred amount reduces significantly from print to print (Figure 1b). When new paste is added to the screen (marked by “*”), *m*_wet_ is initially increased but then decreases again. We have previously shown that the decay of *G′* and *G″* can be influenced by the frequency of the amplitude sweep, but *G″* always exceeds *G′* [3]. By measuring *τ* as a function of the shear rate $\dot{\gamma }$ (not shown), we also verified that both pastes behave as shear-thinning liquids.

For the advanced BSG-paste, both the evolution of *m*_wet_ and *G′* and *G″* differ significantly: For low *τ*, the amplitude of *G′* is higher than for *G″*. Only when a yield stress of about *τ* = 100 Pa is overcome, *G″* exceeds *G′*. While *m*_wet_ still decreases with each print, the decay is much lower and can be stabilized by regularly feeding new material into the screen.

### 3.2. Thin-Film Properties after Thermal Conversion

To analyze the thin-film properties, the refractive index n was determined from spectral ellipsometry. Moreover, chemical-bond formation was analyzed using FTIR.

**Figure 2:**
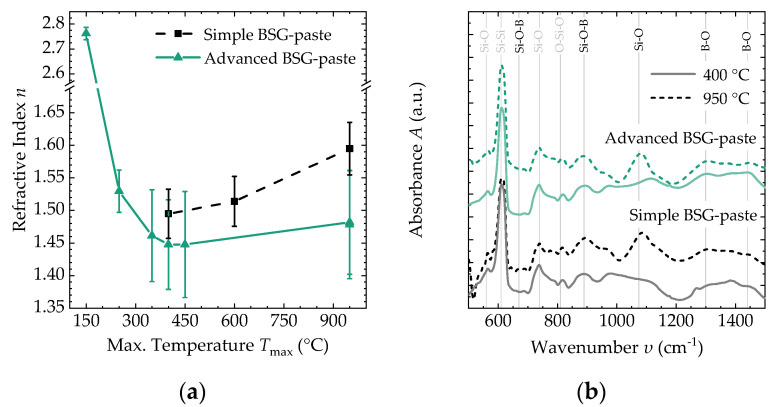
Spectroscopic analysis of obtained thin films. (**a**) Refractive index *n* as a function of the maximum temperature *T*_max_ during thermal treatment; (**b**) absorbance *A* of differently treated films as a function of wavenumber υ indicating the presence of characteristic atomic bonds in the films.

For both materials, n is influenced by the maximum temperature of thermal treatment *T*_max_ (Figure 2a). A strong decrease in n can be found for the advanced BSG-paste when comparing the maximum temperature *T*_max_ = 150 °C to *T*_max_ = 300 °C. At higher temperatures, n first remains at a constant level and then slightly increases at temperatures of around *T*_max_ = 950 °C. For the simple BSG-paste, only the evolution from *T*_max_ = 400 °C to *T*_max_ = 950 °C was investigated. Here also an increase of n is observed. Under all conditions investigated, n is higher for the simple BSG-paste than for the advanced BSG-paste. The values of n under different conditions are summarized in Table 1.

For the silicon substrate, distinct peaks are observed in the infrared spectra at certain wavenumbers *υ* (Figure 2b), (e.g., “Si–Si” at *υ* = 616 cm^−1^ [23]). The signatures of both pastes after thermal treatment are very similar, and no significant differences could be determined in this study. A broad absorption band is found in between *υ* = 1300 cm^−1^ and *υ* = 1400 cm^−1^, which can be associated with different B–O bonds [17,24,25,26,27]. A peak at *υ* = 1100 cm^−1^ occurs after high-temperature treatment with oxygen and can be associated with additional silicon oxidation (Si–O-bond formation) [24]. The interconnection of the SiO_2_ and B_2_O_3_ network, associated with the Si-O-B peaks, is observed as a peak at around *υ* = 900 cm^−1^ [17,24,25,27,28].

### 3.3. Achievable Dopant Concentration

The boron atoms in the dried films were analyzed using ICP-OES after dissolving the BSG layer. When the boron atoms diffuse into the silicon substrate, they can act as dopants, leading to an increase of the hole concentration *p*^+^. This has been measured using ECV.

**Figure 3:**
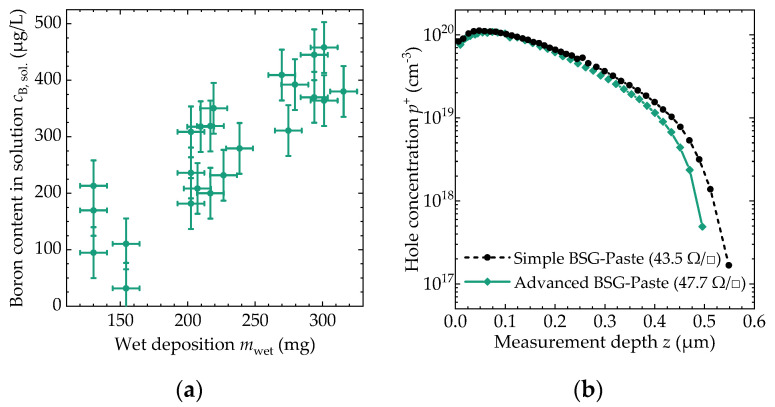
Dopant analysis of the thin films obtained. (**a**) Boron content *c*_B,sol_ determined after dissolution as a function of the amount of paste transferred to the wafer *m*_wet_; (**b**) hole concentration *p*^+^ near the surface of a printed wafer as a function of measurement depth *z* after thermal treatment at a temperature of 950 °C. In the legend, the sheet resistance *R*_□_ of the doped layer is given in brackets.

Samples with the advanced BSG-paste were analyzed to determine the boron content which is present as a dopant reservoir during high-temperature annealing. The boron content found in the solution of the dissolved thin film *c*_B,sol_ is a linear function of the wet deposition, as shown in Figure 3a. When measuring the thin-film thickness using ellipsometry and converting the weight content *c*_B,sol_ into an atomic concentration *ρ*_B_, a rather constant mean value of *ρ*_B_ = 2.5 × 10^21^ cm^−3^ ± 0.4 × 10^21^ cm^−3^ is found. 

The boron atoms can diffuse into the silicon wafer at elevated temperatures. Figure 3b shows the hole concentration profile p^+^ for both BSG-pastes, after a tube-furnace process with a peak temperature of 950 °C, held for 30 min. The dopant profile for both pastes is very similar and the sheet resistance *R*_□_ of the doped layer at the measurement position, given in brackets in the legend, is very close. The distribution of the sheet resistance was also determined across six-inch silicon wafers. The mean value and standard deviation for the simple BSG-paste are *R*_□_ = (42.5 ± 2.6) Ω/□, while the advanced BSG-paste had a sheet resistance of *R*_□_ = (46.4 ± 3.6) Ω/□.

## 4. Discussion

The simple sol-gel synthesis shows non-ideal properties for the use in screen printing. As shown in Section 3.1, any energy put into deformation of the material results in flow as the viscous modulus *G″* exceeds the elastic modulus *G′*, even at the lowest shear stress, *τ*. This can lead to a dripping of paste through the screen mesh. In addition, the wet deposition *m*_wet_ decreases significantly as the sheared paste changes its properties.

With the advanced paste process, a more stable material is created. For low *τ*, the energy is stored in the elastic properties, reflected in the value of *G′*, as expected for a gel. Only after a yield stress is reached, a flow of the material (*G*″ > *G′*) is induced. This material shows a more stable *m*_wet_ during continuous printing.

From sol-gel theory, one can assume that the simple BSG-paste is not fully gelled, explaining some of the short-comings of this paste during long-term printing [29]. The advanced BSG-paste shows higher values of *G*″ and *G′*, which is in line with the longer gelling time used in the sol-gel synthesis. However, as the material incorporates several additives, a direct application of sol-gel theory might not be appropriate.

In the literature [25], the refractive index of sol-gel-derived BSG is in between *n* = 1.47 and *n* = 1.55. As shown in Section 3.2, the refractive indices for the fully dried thin films determined in this study (Table 1) are well within this range. The initial decrease of n at lower temperatures should be correlated with the evaporation of solvents and other additives. The increase for higher temperatures can be associated with additional densification of the thin films, accompanied by a decrease in film thickness. The difference between both materials might hint at a different constitution of the network or a different ratio of the involved atomic species. However, a more conclusive analysis was not possible within this study.

The identified chemical bonds in the material show the formation of a BSG network. As the network is formed by bridging oxygen atoms, no Si–B signals are found in the material. Additionally, no signal of organic residuals is measured, indicating that the additives used to improve the printing properties can be evaporated by sufficient thermal treatment.

The broad absorption band associated with different B–O bonds might hint at differently bound boron atoms. The signals found at higher wavenumbers of up to *υ* ≤ 1440 cm^−1^ might be associated with B–O stretching vibrations in boric acid-like configurations (B(OH)_3_) [17,26]. These might be still present from the used precursors or they occur due to hydrolysis, caused by moisture in the atmosphere [28,30]. Signals at lower wavenumbers hint at more firmly bound B–O.

As shown in Section 3.3, the number of available dopants in the thin film can be used to effectively dope silicon surfaces. In this study, both pastes show very comparable doping properties and very homogeneous doping across the measured wafers. It can therefore be assumed that the different synthesis process and additives used to improve the printing process do not have a significant influence on the use of the BSG-paste as a dopant source. Additionally, the achieved sheet resistances of about *R*_□_ ≈ 50 Ω/□ are sufficient for the fabrication of IBC solar cells [3].

## 5. Conclusions

In this work, we have shown that a more stable screen printing of sol-gel-derived paste can be achieved by adjusting the sol-gel process and by adding suitable additives to the paste. A prolonged gelling can avoid dripping of the paste through the screen, as the elastic modulus dominates at low shear stress. In long-term printing, stable process conditions were found which enable cost-efficient mass fabrication of BSG layers on silicon substrates.

Importantly, the adjusted material is still usable as a silicon dopant source, as desired for the simplified fabrication of high-efficiency IBC solar cells. From the spectroscopic analysis, no significant differences were observed between the derived thin films. The analysis of doping after thermal annealing shows very similar results.

In the future, these processes can be combined with the established screen printing of metallization pastes to realize “all-screen-printed” IBC solar cells or fully passivated “TOPCon” solar cells [18,31] with local, passivating contacts at the front side.

## Figures and Tables

**Table 1:** Refractive indices *n* obtained for two different pastes after thermal treatment at two different temperatures. The given uncertainties are statistical only.

	400 °C	950 °C
Simple BSG-paste	1.51 ± 0.03	1.57 ± 0.04
Advanced BSG-paste	1.45 ± 0.07	1.48 ± 0.08
